# Structure and Chain Dynamics of Self-Healing Telechelic Polymer Networks

**DOI:** 10.1021/acs.macromol.5c01216

**Published:** 2025-09-02

**Authors:** Reidar Lund, Lutz Willner, Olaf Holderer

**Affiliations:** † Department of Chemistry, 6305 University of Oslo, Postboks 1033 Blindern, 0315 Oslo, Norway; ‡ Hylleraas Centre for Quantum Molecular Sciences, University of Oslo, Postboks 1033 Blindern, 0315 Oslo, Norway; § Jülich Centre for Neutron Science (JCNS-1), 28334 Forschungszentrum Jülich GmbH, 52425 Jülich, Germany; ∥ Jülich Centre for Neutron Science (JCNS) at Heinz Maier-Leibnitz Zentrum (MLZ), Forschungszentrum Jülich GmbH, 85747 Garching, Germany

## Abstract

The development of self-healing materials, which are capable of reforming into their original structure following rupture and damage, represents a fascinating and important area of research, with a wide range of potential applications. Telechelic polymers, defined as polymers with functional chain ends such as hydrophobically end-modified polymers, serve as prime examples of systems capable of forming hydrogel networks with transient bonding structures. In this study, we investigate the internal chain dynamics and self-diffusion of hydrogel networks made from telechelic polymers, employing selective contrast variation for small-angle neutron scattering and neutron spin echo spectroscopy. We show that the chain dynamics in the gel follow regular Zimm dynamics without any apparent evidence of restricted motion due to chain connectivity, possibly because the lifetime of the bonds is short. On the other hand, the micellar cores show slow relaxation, reflecting the restricted motion due to the connectivity and crowdedness of the system. The study highlights the decoupling between the slow dynamics of the micellar cores, which play a critical role in the rheological response, and the fast, relatively unconstrained chain dynamics that contribute to their “self-healing” properties. The results provide detailed insight into the multiscale dynamics in hydrogels with transient bonds useful for applications of these types of materials, natural or synthetic.

## Graphical abstract

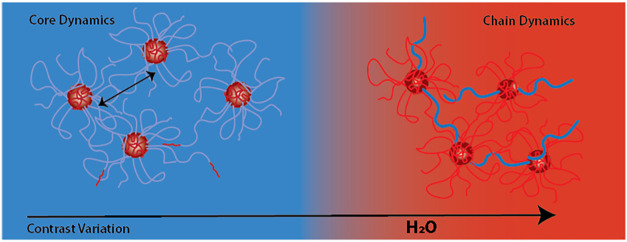

## Introduction

Self-healing materials, i.e., materials that reform into the same structure after suffering rupture and damage, are not only fascinating but also important for many applications. Examples of such networks are supramolecular networks and vitrimers as well as associative and self-assembling polymers where specific hydrogen-bonding, ionic complexation, or hydrophobic interactions lead to connected networks.^1,2^ Telechelic polymers, which are polymers with functional chain ends such as hydrophobically end-modified polymers, are fascinating examples of systems capable of forming hydrogel networks with transient bonding structures.^3,4^ Telechelics, whose terminal groups have the capacity to form intramolecular and intermolecular bonds, may self-assemble in micellar-like entities. Here, the polymers can “cross-link” intermolecularly to form a micellar network that progressively develops at higher concentrations (see the illustration in Figure 1).

**Figure 1:**
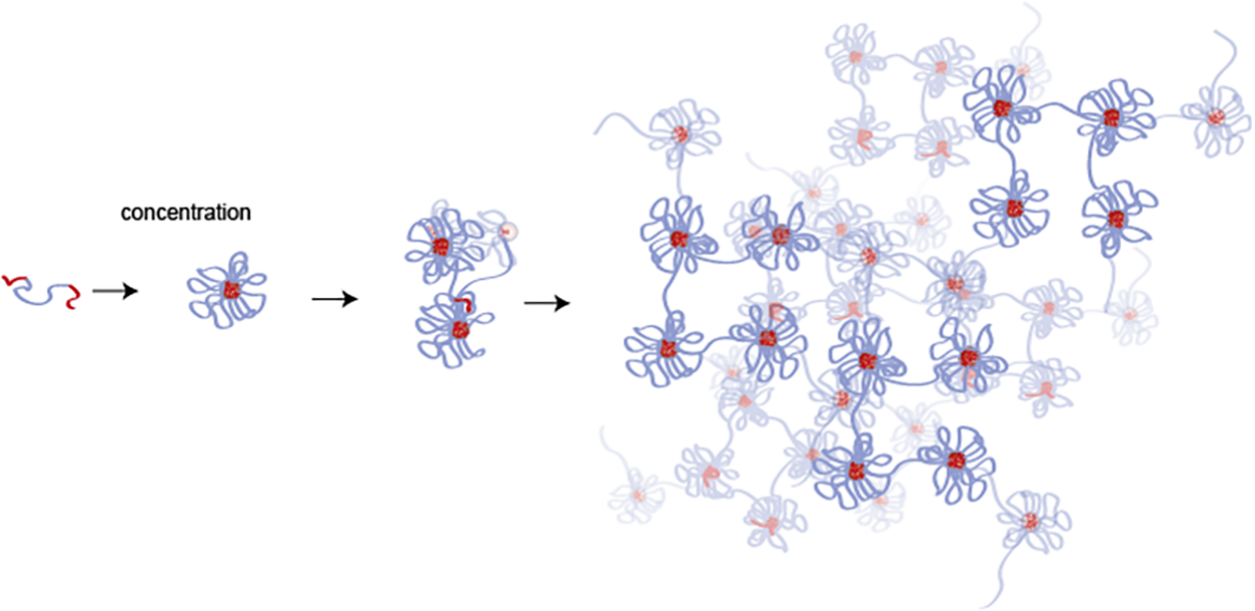
Illustration of the self-assembly and sol–gel transition of telechelic polymers. With increasing concentration, telechelic polymers self-assemble into micelles that interconnect and finally form nanostructured percolated hydrogels.

Since hydrogels formed by associative polymers such as telechelics are built up from noncovalent bonds, the connectivity is transient, i.e., each bond has a finite lifetime.^3^ These gel networks with temporal bonds exhibit desirable rheological characteristics such as shear-thinning and fast recovery, which is important for many applications^5–8^ such as injectable hydrogels for biomedical applications, in agriculture for the distribution of fertilizers,^9^ scaffolds for tissue regeneration, etc. However, as a consequence of the rather rich dynamics on various length/timescales, the rheological properties in these systems can be rather complex and dependent on a variety of factors.^10^ In addition to the usual variables, i.e., molecular weight, concentration, etc., the dynamics is largely dependent on the functionality and density of the functional groups, bonding energy, and potentially, the nature of the self-assembled nanostructure formed. An important concept to understand the dynamics of associating polymers is the sticky Rouse model first proposed by Baxandall^11^ and later further developed by Leibler, Rubinstein, and Colby,^12,13^ and the transient network model by Tanaka and Edwards.^14^ These models extend the classical Rouse model to account for transient cross-links and interactions caused by “sticky” often hydrophobic groups. The relaxation behavior is governed by the lifetime of these transient interactions, leading to a distinct viscoelastic response. Linear rheological experiments of telechelic polymers have shown that the chain relaxation at low frequencies is dominated by breaking and reforming “sticky” bonds, resulting in a characteristic peak of the loss modulus *G* ^“^ that directly reflects the lifetime of the bonds.^6^

Most experimental studies related to telechelic/associative polymer gels have been devoted to their intriguing rheological properties.^3,15^ There is considerable less work done using experimental methods that are capable of resolving the overall chain dynamics. The dynamics of telechelic polymer melts have been studied using dielectric spectroscopy, showing that both the local, segmental, and the global, end-to-end chain vector (“normal mode”) relaxation are slowed down due to chain association.^7^ Interestingly, it was found that the bond lifetime and terminal relaxation time differ by 1–4 decades depending on molecular weight and associating strength.^16^ This was attributed to the restricted network dynamics that leads to longer effective lifetime of the reversible bonds (“renormalized” relaxation time).^13^ NMR relaxation experiments have also provided insight into segmental dynamics of telechelic polymers and its relation to their rheological properties.^17,18^ The results show that in this case, the terminal rheological relaxation is governed by chain relaxation rather than micellar networks, implying significant chain mobility despite the transient cross-links. However, by these methods, the global chain dynamics and spatially resolved modes are not accessible. In contrast, neutron spin echo (NSE) spectroscopy has the ability to directly measure dynamic correlations over a very wide range of time (picoseconds to microseconds) and length scales (Ångström to nanometers), providing detailed spatial and temporal resolution that is particularly useful for studying large-scale molecular relaxations as well as local motion of polymers.

NSE has been used to investigate telechelic polymer networks built up from polymers bearing complementary hydrogen-bonding end-groups.^19^ The results show that the polymers display a Rouse-like motion but with a modified mode spectrum. The dynamics of transient dense protein networks has also been studied showing evidence for cooperative diffusive modes, which depends on the cross-link density.^20^ The transition from Zimm segmental dynamics to collective modes has been analyzed in well-ordered PEO gels.^21^ Homogeneously and heterogeneously cross-linked microgels show density fluctuations and segmental relaxations.^22^ A mixture of permanent and transient cross-links has been analyzed with rheological methods and neutron scattering.^23,24^ The aggregation behavior and segmental dynamics of diblock- and triblock-copolymers made from poly­(styrene) (PS) and poly (*N*-isopropylacrylamide) (PNIPAM) were studied with neutron scattering (SANS, NSE)^16,25^ with a focus on the thermoresponsive behavior of the PNIPAM shell. In this work, we investigate the chain dynamics of hydrogels formed by well-defined telechelic polymers. The polymer system consists of well-defined poly­(ethylene oxide) (PEO) end-functionalized with *n*-alkyl groups of defined length.^26^ As shown in previous work, the system assumes the form of clustered micelles at low concentrations, whereas percolated hydrogel networks are formed as the concentration is increased.^6,26^ The bond lifetime, i.e., the residence time of a sticker, is critically dependent on the n-alkyl length.^27^ At low concentrations, it was found that micelles formed by telechelic PEOs exhibit a collision-induced two-step exchange mechanism where one chain end is released first allowing for transfer to another micelle, but only upon direct contact.

For denser concentrations, a comparison between time-resolved small-angle neutron scattering (TR-SANS) and linear oscillatory shear rheology showed that network relaxation can be directly related to the exchange kinetics, which dictates the bond lifetime. In this work, we investigate chain dynamics in detail, specifically addressing the effect of constraints imposed by the transient network structure. We investigate the internal chain dynamics and self-diffusion of hydrogel networks using selective contrast variation and neutron spin–echo (NSE) spectroscopy, with the aim of deciphering the contributions leading to their complex rheological response. Using H/D contrast variation, we can measure the chain and micellar core dynamics within gel networks separately. The results indicate that the center-of-mass diffusion of the network nodes, specifically the micellar cores, is exceedingly slow and is significantly constrained by the transiently cross-linked chains. In contrast, individual chains exhibit regular Zimm dynamics. This behavior likely reflects the rapid exchange kinetics and relatively short residence times within the cores, which diminish the constraints and result in dynamics similar to those of “free” chains.

## Experimental Section

### Polymer Synthesis

The synthesis of the polymers used in this study was accomplished by a two-step procedure as described in more detail in previous publications.^6,26,28^ In a first step, C_16_ hydrophobically monofunctionalized poly­(ethylene oxide) (PEO) polymers were synthesized by living anionic polymerization of ethylene oxide (EO) using 1-hexadecanol (C_16_-H_33_-OH) and the corresponding potassium salt (C_16_-H_33_-O^–^K^+^) in an 80:20 mixture as an initiator. The living polymers were terminated with acetic acid, leading to a hydroxy group in the terminal position. For H/D contrast variation experiments, two differently labeled polymers were prepared: d-C_16_-h-PEO5-OH and h-C_16_-d,h-PEO5-OH with 5 kg/mol as the target molecular weight. h,d-PEO denotes a PEO polymer with a random distribution of h- and d-EO units along the polymer chain, prepared from a mixture of d- and h-EO monomers with 72 mol% of the deuterated compound. The molar characteristics of the polymers were determined by size exclusion chromatography (SEC) relative to narrowly distributed PEO standards. Exact molar masses were calculated by comparing the elution volumes, *V*_e_, with *V*_e_, of h-C16-h-PEO5-OH. h-C16-h-PEO5-OH were adopted from an earlier study.^26^ The thus obtained molar masses are 5065 kg/mol for d-C_16_-h-PEO5-OH and 5450 kg/mol for h-C_16_-d,h-PEO5-OH, taking into account the higher *M*_n_-values due to partial deuteration. The dispersity, *M*_w_/*M*_n_, was smaller than 1.03 for both polymers. In a second step, α/ω-difunctionalized (telechelic) polymers, d-C_16_-h-PEO10-d-C_16_ and h-C_16_-d,h-PEO10-h-C_16_, were prepared via intermolecular condensation of the terminal hydroxy groups of the monofunctionalized polymers. The condensation was carried out by the reaction with tosyl chloride in the presence of solid potassium hydroxide. The reaction products were fractionated with chloroform/heptane as the solvent/nonsolvent pair. In this way, we obtained almost pure telechelic h-C_16_-d,h-PEO10-h-C_16_ with a residual uncoupled monofunctional polymer of 2–3% as proved by SEC. In the case of d-C_16_-h-PEO10-d-C_16_, the coupling reaction was less successful. After fractionation, the yield of the product was too low to be able to continue with experiments. This fraction was just used for characterization only. Instead, we have used combined fractions that still contained 19% of the parent material (relevant SEC data are shown in the SI) to carry out NSE and SANS experiments. Since no degradation or cross-linking occurred during the coupling reaction, the molar masses of the two telechelic polymers were assumed to be twice that of the parent monofunctionalized materials, i.e., 10.1 kg/mol for d-C_16_-h-PEO10-d-C_16_ and 10.9 kg/mol for h-C16-h,d-PEO10-h-C16, respectively. It should be mentioned that SEC data show almost exactly the same elution volumes for the two telechelic polymers (see corresponding SEC data in the SI), reflecting equal degrees of polymerization. This is an essential condition for applying the zero average contrast technique to elucidate the single chain structure and dynamics in the hydrogel networks. In addition, two PEO homopolymers h-PEO10 and d-PEO10 were kindly provided by Dr. Jürgen Allgaier (FZ-Jülich GmbH) as reference materials. These homopolymers were prepared with the bifunctional initiator tetraethylene glycol metalated with 14% potassium. The molar mass was *M*_n_ = 9.29 kg/mol for h-PEO10 and 10.9 kg/mol for the deuterated material as measured by SEC relative to PEO standards.

### Sample Preparation

All samples for SANS and NSE experiments were prepared at a polymer volume fraction of ϕ = 11%. Core contrast sample h-C_16_-d,h-PEO10-h-C_16_ in D_2_O/H_2_O with Φ_*D*_2_ *O*_ = 88.2% (matches d,h-PEO10). Single chain contrast sample: d-C_16_-h-PEO10-d-C_16_/d-C_16_-h-PEO5-OH­(19%) + h-C_16_-d,h-PEO10-h-C_16_/h-C_16_-d,h-PEO5-OH (19%) in D_2_O/H_2_O with Φ_D_2_O_ = 53.3% (zero average contrast condition for PEO chains). For the preparation of the single chain contrast, the almost pure telechelic polymer h-C_16_-d,h-PEO10-h-C_16_ was blended with h-C_16_-d,h-PEO5-OH to achieve similar compositions of difunctional/monofunctional for the two differently labeled materials. Homogeneous blends were obtained by dissolving in chloroform and drying in vacuum. Finally, polymer solutions of 11% volume fraction were prepared in the corresponding H_2_O/D_2_O water mixtures. Hydrogel samples were obtained by a centrifuge. Vials were turned several times and heated above 50 °C, where the hydrogels became a liquid. In this way, homogeneous and clear gels were obtained and filled bubble-free into quartz glass Hellma cells for measurements.

### SANS

SANS experiments were performed at the KWS-2 instrument located at Heinz-Meier Leibnitz Zentrum (MLZ) in Garching, Germany.^29^ Sample-to-detector distances of 2 and 8 m with a collimation length set to 8 m and a neutron wavelength of 7 Å with a wavelength spread of Δλ/λ = 20% were used to cover a *Q*-range from 0.01 to 0.28 Å^–1^, where *Q* = |*Q⃗*| = (4π sin­(θ/2))/λ is the modulus of the momentum transfer and θ is the scattering angle.

### Data Modeling

As previously shown,^26^ telechelic C_*n*_-PEO-C_*n*_ polymers form a core–shell structure where the *C*_*n*_-alkyl chains form the micellar “core” and the PEO assumes the surrounding “shell”. Since there are alkyl groups located at each chain end of PEO, the system tends to cross-link where a fraction of the chain ends are buried in the core of different micelles, causing the formation of a transient polymer network. To describe the scattering from such a system, we have to start with a core–shell model for the form factor, *P*(*Q*), describing the individual micelles constituting the network (c.f. Figure 1) and implement the spatial correlation of the entities using a structure factor, *S*(*Q*). For the data under “core contrast”, we observe rather well-developed Bragg peaks with a high degree of order where the micellar cores are able to order into a mesoscopic crystal. We therefore employed a combined form and structure factor model for a mesoscopic crystal structure previously reported in detail by Förster et al.,^30^ which, adapted to our systems, can be described as follows

1 $$I\left(Q\right)=\phi \cdot \left(1-\phi \right)(\rho_{C16}-\rho_{0}{)}^{2}\cdot N_{\mathrm{agg}}\cdot V_{C16}\cdot P\left(Q\right)\cdot \left(1+\beta \left(Q\right)\left(Z_{0}\left(Q\right)-1\right)\cdot \mathrm{DWF}\left(Q,\sigma_{D}\right)\right)$$

where *N*_agg_ is the aggregation number, $V_{C_{16}}=\frac{M_{C_{16}}}{d_{C_{16}}N_{\mathrm{Avo}}}$ is the molecular volume of C16, given by its molecular weight *M*_C_16__ = 45 g/mol and density *d*_C_16__ = 0.77 g/mL. *N*_Avo_ is Avogadro’s number, and ρ_*i*_ represents the scattering length density, where *i* = C_16_ for C16 or *i* = 0 for the solvent. To account for the reduction in scattering intensity due to thermal vibrations in the crystal lattice, we included the “Debye–Waller factor”

$$\mathrm{DWF}\;\left(Q,\sigma_{D}\right)=\exp\left(-Q^{2}\cdot \sigma_{D}^{2}\right)$$

This expression assumes that the crystal lattice distortions can be described as the Gaussian distribution of isotropic displacements around the center-of-mass, characterized by a width σ_*D*_. Finally, $\beta \left(Q\right)=\frac{\langle A\left(Q\right)\rangle^{2}}{\left\langle A{\left(Q\right)}^{2}\right\rangle }$ , where *A*(*Q*) is the scattering amplitude of the particular particle.

The form factor, *P*(*Q*) = ⟨*A*(*Q*)^2^⟩, for each geometry can be approximated by

2 $$A\left(Q\right)=\frac{3\left(\sin\left(Q\cdot R_{c}\right)-Q\cdot R_{c}\cos\left(Q\cdot R_{c}\right)\right)}{{\left(Q\cdot R_{c}\right)}^{3}}\cdot \mathrm{DW}\left(Q,\sigma_{\mathrm{int}}\right)$$

In order to take into account interfacial roughness of the micellar cores, we have included another “Debye–Waller” factor DW­(*Q*, σ_int_) = exp (−*Q* ^2^ σ_int_^2^). This arises for a sphere convoluted with a Gaussian function describing the outer roughness given by the width σ_int_ ^31^ In addition, we averaged the form factor over a Gaussian distribution function describing the polydispersity in the size of the cores, *R*_c_,i.e.

3 $$f\left(r,\sigma_{R}\right)=\frac{1}{\sigma_{R}\sqrt{2\pi }}\exp\left(\frac{-{\left(r-R_{c}\right)}^{2}}{2\sigma_{R}^{2}}\right)$$

where σ_R_ thus characterized the width of the distribution.

The ideal structure factor, *Z*_0_(*Q*), can simply be written as a sum of peak functions representing the reflections characteristic of the crystal structure as

4 $$Z_{0}\left(Q\right)=\frac{c}{4a^{3}\cdot Q^{2}}\sum_{h,k,l}m_{\mathrm{hkl}}f_{\mathrm{hkl}}^{2}L_{\mathrm{hkl}}\left(Q\right)$$

where 1 < *d* < 3 is the dimensionality, *m_hkl_* is the multiplicity, and *f_hkl_* is a symmetry factor of the reflections. *c* is a correction factor to ensure that Porod invariant is fulfilled,^30^ where *h*, *k*, and *l* are the Miller indices of the particular crystal structure. *Q*_*hkl*_ satisfies the following conditions for each geometry

5 $$Q_{\mathrm{hkl}}=\frac{2\pi \sqrt{\left(h^{2}+k^{2}+l^{2}\right)}}{a_{\mathrm{lat}}}$$

Here, (*h*, *k*, *l*) takes the well-known discrete values characterizing the crystallographic planes of the particular crystal, which for the bcc structure relevant for this work take the values (110), (200), (211), (220), (310), etc.^30^

To describe the peak reflections, the following parameterization of the peak functions suggested by Burger-Mischa and Förster et al. (see ref^30^ for details) was used

6 $$L_{\mathrm{hkl}}\left(Q\right)=\frac{2}{\pi \delta }\prod_{n=0}^{n_{\max}}{\left(1+\frac{4\gamma_{\nu }^{2}\cdot x^{2}}{{\left(n+\nu /2\right)}^{2}\cdot \pi^{2}\delta^{2}}\right)}^{-1}$$

where *x* = (*Q* – *Q*_*hkl*_) and $\gamma_{\nu }=\pi^{1/2}\frac{\Gamma \left(\nu +1\right)/2}{\Gamma \left(\nu /2\right)}$ where Γ­(*y*) is the γ function. The peak width, δ, is related to the domain size by ξ = 2π/δ.

In the final fits, the shape parameter was fixed to *ν* = 50, and the aggregation number was allowed to vary, resulting in a value of *N*_agg_ = 15, ± 2. Since the micellar cores are water-free,^26,32^ the micellar core radius can be constrained by $R_{c}={\left(\frac{3N_{\mathrm{agg}}\cdot V_{C_{16}}}{4\pi }\right)}^{1/3}$ .

#### Analysis of Single Chain Scattering

To describe polymer scattering, either PEO homopolymers or the gels under “single chain contrast”, we used the following expression describing free chain scattering

7 $$I\left(Q\right)=\phi \cdot \left(1-\phi \right)\cdot \Delta \rho_{\mathrm{mean}}^{2}\cdot M_{\mathrm{poly}}/d_{\mathrm{poly}}/N_{A}\cdot P{\left(Q\right)}_{\mathrm{poly}}$$

where ϕ is the volume fraction, Δ*ρ*_mean_ is the mean contrast, *d*_poly_ is the density, *M*_poly_ is the molecular weight, *N*_A_ is the Avogadro’s number, and *P*(*Q*)_poly_ is the form factor of single chains. For the latter, we considered three form factors. First, we employed the well-known Debye function for Gaussian chains

8 $$P{\left(Q\right)}_{\mathrm{poly}}^{\mathrm{Debye}}=\frac{2\left(\exp^{-x}-1+x\right)}{x^{2}}$$

where *x* = *Q* ^2^ *R*_g_^2^

To account for long-range excluded volume effects of chains in solution, we considered the Beaucage form factor

9 $$P{\left(Q\right)}_{\mathrm{poly}}^{\mathrm{Beau}}=\exp\left(-Q^{2}R_{g}^{2}/3\right)+\frac{d_{f}}{{\left(Q\cdot R_{g}\right)}^{d_{f}}}\Gamma \left(d_{f}/2\right)\cdot {\left(\frac{\mathrm{erf}{\left(Q\cdot R_{g}/\sqrt{6}\right)}^{3}}{Q}\right)}^{d_{f}}$$

where *R*_g_ is the radius of gyration, and *d*_f_ is the fractal dimension (*d*_f_ = 2 and 1.7 for theta solvent and good solvent, respectively). Γ­(*x*) is the γ function, and erf­(x) is the error function.

And the generalized Debye function

10 $$P{\left(Q\right)}_{\mathrm{poly}}^{\mathrm{Gen}.\mathrm{Debye}}=\frac{1}{\nu \cdot U^{1/2\nu }}\cdot \Gamma_{\mathrm{inc}}\left(\frac{1}{2\nu },U\right)-\frac{1}{\nu \cdot U^{1/\nu }}\cdot \Gamma_{\mathrm{inc}}\left(\frac{1}{\nu },U\right)$$

where $U=\frac{Q^{2}R_{g}^{2}\left(2\nu +2\right)\left(2\nu +1\right)}{6}$ and ν is the Flory exponent related to the fractal dimension as *d*_f_ = 1/ν. Γ_inc_(*x*, *U*) = ∫_0_^*U*^ exp­(−*t*)*t* ^*x*–1^d*t* is the incomplete γ function.

### NSE

Neutron spin echo (NSE) spectroscopy provides the highest possible energy resolution of all neutron spectroscopy techniques. This is achieved by encoding the velocity change during quasi-elastic or inelastic scattering in the beam polarization by a spin echo sequence on the polarized neutron beam. The normalized polarization is directly the intermediate scattering function *I*(*Q*, *t*) = *S*(*Q*, *t*)/*S*(*Q*, *t* = 0), which is the Fourier transform of the scattering function S­(Q,E) into the time domain. The instrumentally achievable Fourier time is of the order of some 100 ns, and the range of momentum transfer corresponds well to molecular length scales (i.e., to the *Q*-range of an accompanying SANS experiment). Details of the NSE technique can be found, e.g., in refs 33,34

#### Experimental NSE Setup

Neutron spin echo (NSE) experiments were performed at the J-NSE instrument with normal conducting copper main coils^35^ operated by the Jülich Centre for Neutron Science (JCNS) at Heinz Maier-Leibnitz Zentrum (MLZ) in Garching. A wavelength of λ = 8 Å has been used throughout the experiment to cover a Fourier time range from 0.1 to 40 ns and a *Q*-range of 0.05–0.15 Å^–1^.

#### Data Modeling of the NSE Data

The intermediate scattering function *I*(*Q*,*t*) is a correlation function, which can be modeled with coarse-grained polymer models. Diffusive components just decay with a *Q* ^2^-dependent exponential decay, *I*(*Q*, *t*) = e^(−*DQ* ^2^ *t*)^, with the Stokes–Einstein diffusion constant D. Deviations from the *Q* ^2^-dependence and from the simple exponential decay can be described by a stretching exponent β (i.e., *I*(*Q*, *t*) = e^–(*Γt*)^β^^, which is characteristic of, e.g., polymer melts (β = 0.5) or polymers in solution (β = 0.84)).^36^ A very slow contribution with dynamics far slower than the time window of the experiment results in an apparently elastic plateau to which the correlation function decays. A more detailed analysis with the full Rouse or Zimm model as described in ref^36^ allows us to model mode restrictions or geometrical restrictions and provides more flexibility as the simplified equations. The dynamics measured in NSE is largely dominated by the coherent dynamics of the polymer chains or the core (in core contrast) at low *Q*. Incoherent scattering mainly coming from the solvent has been subtracted by proper background measurements (with individual background samples for each composition) and can be neglected.

## Results and Discussion

### Structure of the Nanostructure Hydrogels

Based on previous studies of dilute C_*n*_-PEO-C_*n*_ telechelic polymers, core shell structures are formed where the alkyl chains form the cores and the PEO constitutes the micellar shell. Because the alkyl chains may connect two neighboring micelles or loop back into its own core, the system consists of “flower-like” micelles and interconnected micellar clusters.^26^ In Figure 1, the self-assembly of these telechelic polymers is depicted, showing the progression of clustered micelles at low concentrations to interconnected micellar gels as the concentration is increased. In this work, we address the chain dynamics in the gel state and choose a concentration of ϕ = 11%, which is well above the overlap concentration. The upper limit of the overlap concentration, corresponding to a flower-like micellar conformation, can be estimated from previously reported data^26^ to be ϕ* ≃ (3*N*_agg_ · *V*_poly_)/(4*πR*_*m*_^3^) = 5–6%, where we have used an aggregation number of *N*_agg_ = 15, a micellar radius of 95 Å, and a molecular volume of a single polymer, *V*_poly_ = 15200 Å^3^.

As illustrated in Figure 2, two contrast conditions were considered to accurately extract the structure and individual contributions to the dynamics using neutron scattering. First, a “core contrast” is found, where the coherent scattering of the micellar cores is visible rendering the motion of the micellar center of mass visible. Here simply h-C_16_-d,h-PEO10-h-C_16_ polymers were dissolved in D_2_O/H_2_O with Φ_D_2_O_ = 88.2% to match out all residual scattering of the partially deuterated PEO chains. Second, we employed a “single chain contrast” where h-C_16_-d,h-PEO10-h-C_16_ polymers were mixed with d-C_16_-h-PEO10-d-C_16_ polymers in a 1:1 ratio and dissolved in D_2_O/H_2_O with Φ_D_2_O_ = 53% such that 1/2­(ρ_*d*,*h*–PEO_ + ρ_*h*–PEO_) = 1/2­(ρ_*d*–C_16__ + ρ_*h*–C_16__) = ρ_0_. This *zero average contrast* (ZAC) only renders the scattering of individual chains visible, allowing us to study the chain conformation and polymer dynamics of the gel.

**Figure 2:**
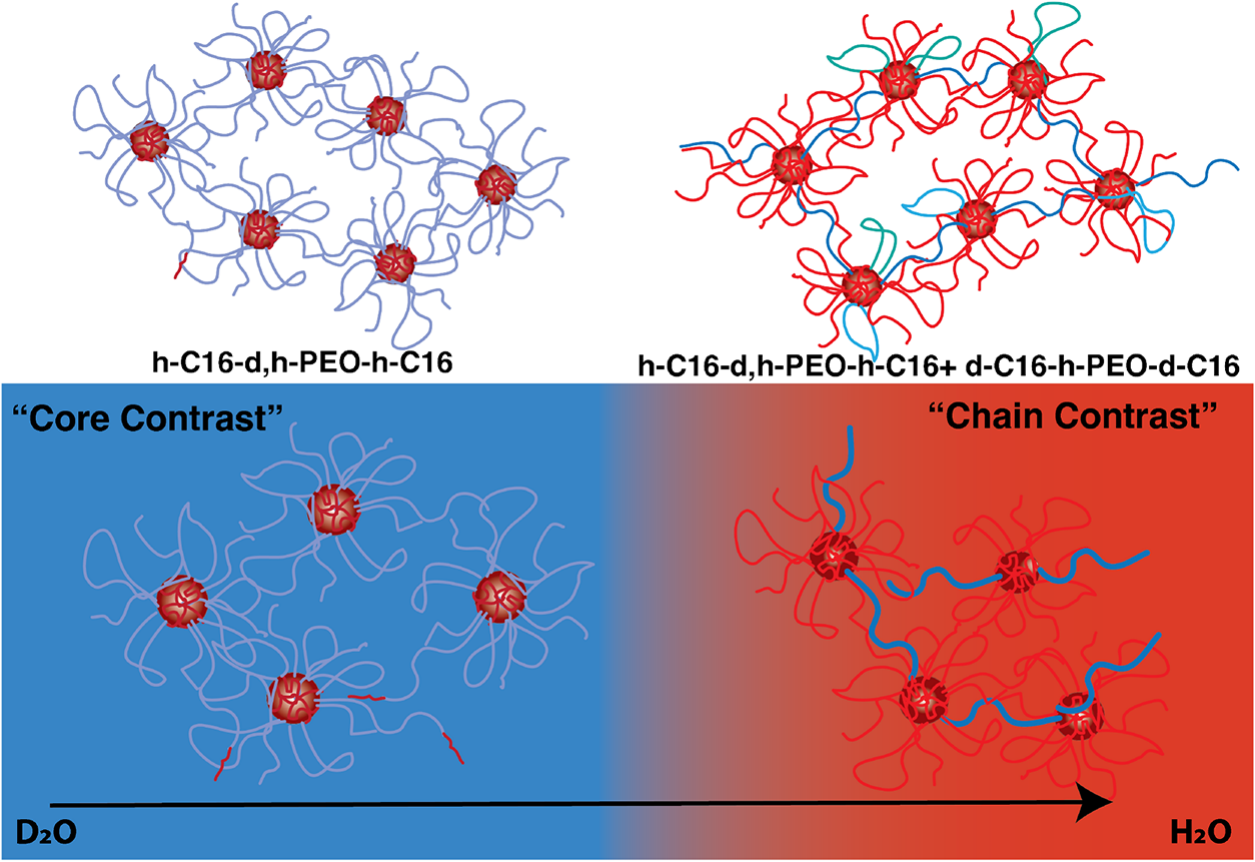
Illustration of contrast variation for neutron scattering experiments. Core contrast (cores are visible) is obtained by h-C_16_-d,h-PEO10-h-C_16_ in D_2_O/H_2_O, Φ_D_2_O_ = 88.2% thereby matching out the partially deuterated PEO. Single chain contrast (bridging PEO chains are visible) is obtained by a blend of two polymers: h-C_16_-d,h-PEO10-h-C_16_ and d-C_16_-h-PEO10-d-C_16_ in D_2_O/H_2_O, Φ_D_2_O_ = 53%, which corresponds to zero average contrast (ZAC) conditions, rendering the individual chains visible. See the text and the Sample Preparation section for additional details.

The SANS data for the two contrast conditions are given in Figure 2A. As expected, the two contrast conditions give distinctly different scattering patterns. The data for the single chain contrast resembles the scattering from a typical polymer chain with a plateau at low *Q* (Guinier regime) followed by an ∼*Q* ^–2^ slope at high *Q*. This is confirmed by a form factor analysis where we used three different form factors for single chains; the Debye model for random (Gaussian) chains, the Beaucage model,^37^ and the generalized Debye model^38^ to account for excluded volume effects. The results, described in the SI, show slight deviations from the ideal Debye model, whereas both excluded volume models provided very good fit descriptions with essentially the same results.

The data for the “core contrast”, on the other hand, display a very different pattern with several features including Bragg-like correlation peaks indicating ordering and a shoulder at high *Q* that reflects the micellar core scattering. In order to describe the data, a combined form/structure factor needs to be included. A fit using the approach described in the Data Modeling section (eqs 3–11) reproduces the data rather well and provide quantitative structural parameters. From the fit, we obtain a core radius of about *R*_c_ = 18 ± 2 Å with 13% polydispersity and a unit cell dimension of 187 ± 2.5 Å. The latter translates into a nearest-neighbor distance of $d=\sqrt{3a}/2$ = 161 Å. The other fit parameters were determined to be σ_int_ = 2.6 ± 0.5 Å, $\frac{\sigma_{D}}{a_{\mathrm{lat}}}$ = 0.5, $\frac{\sigma_{R}}{R_{c}}$ = 0.4, and a domain size of ξ = 153 ± 20 Å. The latter small domain size and rather large disorder indicate a repetitively distorted lattice, consistent with the broad peaks observed in the data.

In Figure 3B, the scattering data for the PEO polymers in the hydrogel (single chain contrast) are compared to those for the equivalent homopolymers. As seen, apart from the intensity, the data look very similar, and the shape of the curves are the same. From the data, we obtain the gyration radii, *R*_g_, of 44 ± 3 and 40 ± 3 Å for the polymer on the gel (single chain contrast) and the homopolymer, respectively. In both cases, we also find a similar apparent fractal dimension of *d*_f_ ≈ 1.9. We can thus conclude, in particular considering the difficulties in accurate subtraction of incoherent scattering, that the chain conformation is largely unaffected by the gel formation and core domains. This is quite intriguing, in particular, considering that the end-to-end distance of the chains, *R*_ee_ ≈ √6*R*_g_ = 98 Å, is smaller than the nearest-neighbor distance, *d* ≈ 160 Å. However, this might be understood in terms of fast exchange kinetics, such that at least one chain end is practically unattached to the anchoring core on average at any given time. From previous results where we have studied the exchange kinetics of telechelics in a dilute solution, we have shown that the system follows a two-step mechanism where one chain end is released and complete transfer of the complete chain only can be achieved via a subsequent collision with another micelle.^28^ Another study showed that the rheological response of similar telechelic hydrogels, more specifically, the loss modulus in linear oscillatory shear experiments, can be directly related to the lifetime of the bond, i.e., the exchange kinetics. For the rather short C_16_ chain end here, we expect the exchange kinetics to be exceedingly fast (≪ seconds). From a previous work,^39^ we can estimate the activation barrier for molten alkyl-PEO micelles as *E*_a_ = [7.6*n* – 91] kJ/mol, where *n* is the number of carbons. This gives *E*_a_ = 30.6 kJ/mol = 12 *k*_B_ *T* at 298 K. This value seems rather large, but calculating the characteristic rate of exchange using τ = τ_0_ exp (*E*_*a*_/*RT*) using a fundamental time scale of τ_0_ of approximately 1 ns for the C_16_ chain, we obtain τ = 0.2 ms. It should be noted that this may be considered an upper limit since the conformational entropy, which has not been taken into account here, will decrease the exchange time further.^39^ This translates into an average lifetime of single polymer with a micelle, τ_life_, of ≈ *N*_agg_ · τ = 0.2 · 15 = 3 ms.

**Figure 3:**
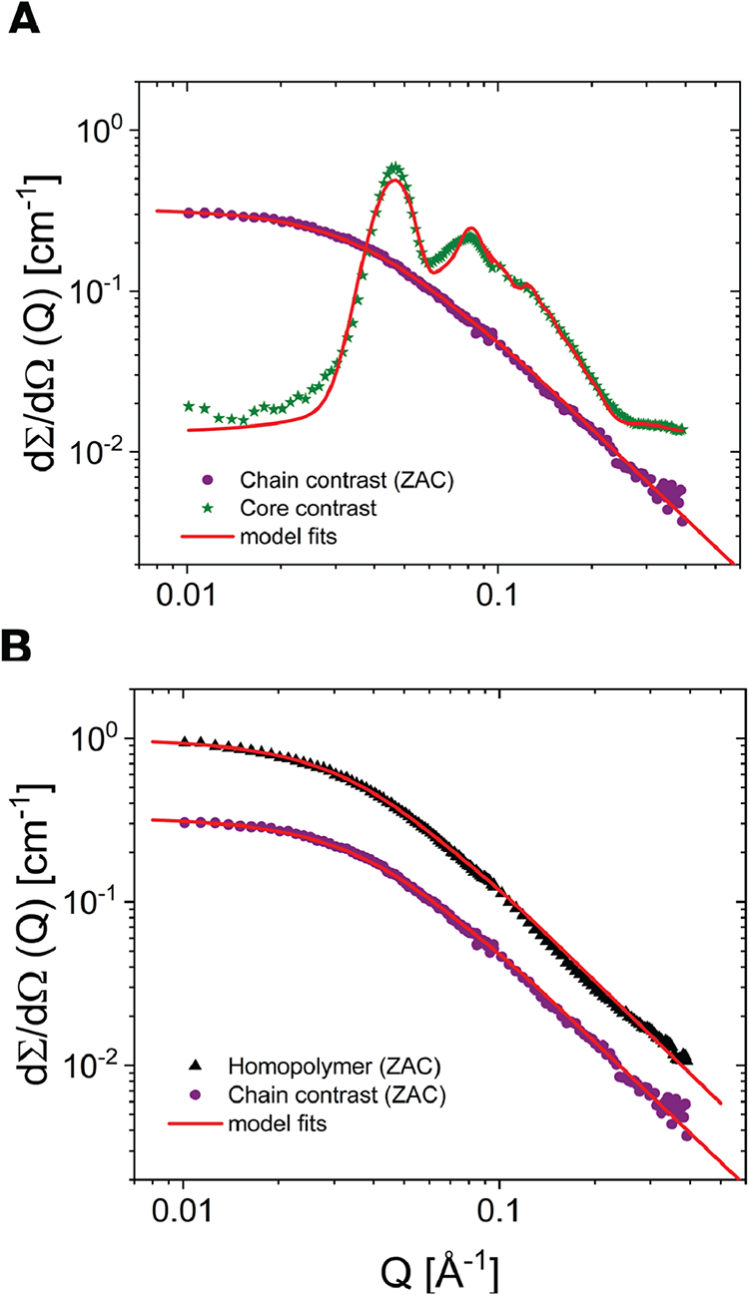
Small-angle neutron scattering data of (A): h-C_16_-d,h-PEO-h-C_16_ 11% in D_2_O/H_2_O, Φ_D_2_O_ = 88.2% (“core contrast”) and d-C_16_-h-PEO10-d-C_16_/h-C_16_-d,h-PEO-h-C_16_ 11% in D_2_O/H_2_O, Φ_D_2_O_ = 53% (“single chain contrast”). Solid lines correspond to the best fit using the Beaucage model for single chains and a combined form/structure factor model for spheres packed in a body-centered cubic (bcc) crystalline structure. (B): Single chain contrast SANS data revealing the chain conformation in the hydrogel and for comparison scattering data of a reference h-PEO10/d-PEO10 11% in D_2_O/H_2_O mixture with zero average contrast composition (ZAC).

In order to understand the dynamics in more detail, NSE experiments were performed for each of the contrasts of the hydrogel as well as the reference PEO homopolymer at the same concentration.

### Dynamics

The reference PEO homopolymer in solvent, the gel in core contrast, and the gel in chain contrast are first evaluated with a stretched exponential function. In core contrast, the low *Q* region is affected by the structure factor (the relaxation time follows S­(*Q*)), but with times of the order of several hundreds of nanoseconds, which cannot be determined very precisely in the time range of this experiment of 40 ns. The fit analysis was done as described in the Data Modeling section, with a stretched exponential function of the form *S*(*Q*, *t*)/*S*(*Q*, 0) = exp­(−(*Γt*)^β^), where an effective diffusion coefficient can be obtained from the relaxation rate Γ with *D*_eff_ = Γ/*Q* ^2^ in the case of β = 1 or with a correction factor *D*_eff_ = Γ/*Q* ^2^β/(Γ_*s*_(1/β)), where Γ_*s*_ is the -γ function, valid for β ≤ 1. Figure 4 shows the intermediate scattering functions of PEO homopolymer in solution, the core contrast, and the chain contrast samples, fitted with a stretched exponential function as a generic model. The PEO homopolymer dynamics follows very well the stretched form for a polymer chain in solution as described with the Zimm model with an exponent β = 0.83 ± 0.06, which is characteristic for this model. Similarly, the chain contrast dynamics follows a stretched exponential function, although due to the statistics with larger errors (β = 0.7 ± 0.2). The effective diffusion coeffecient plotted as a function of *Q* for all three samples are given in Figure 5.

**Figure 4:**
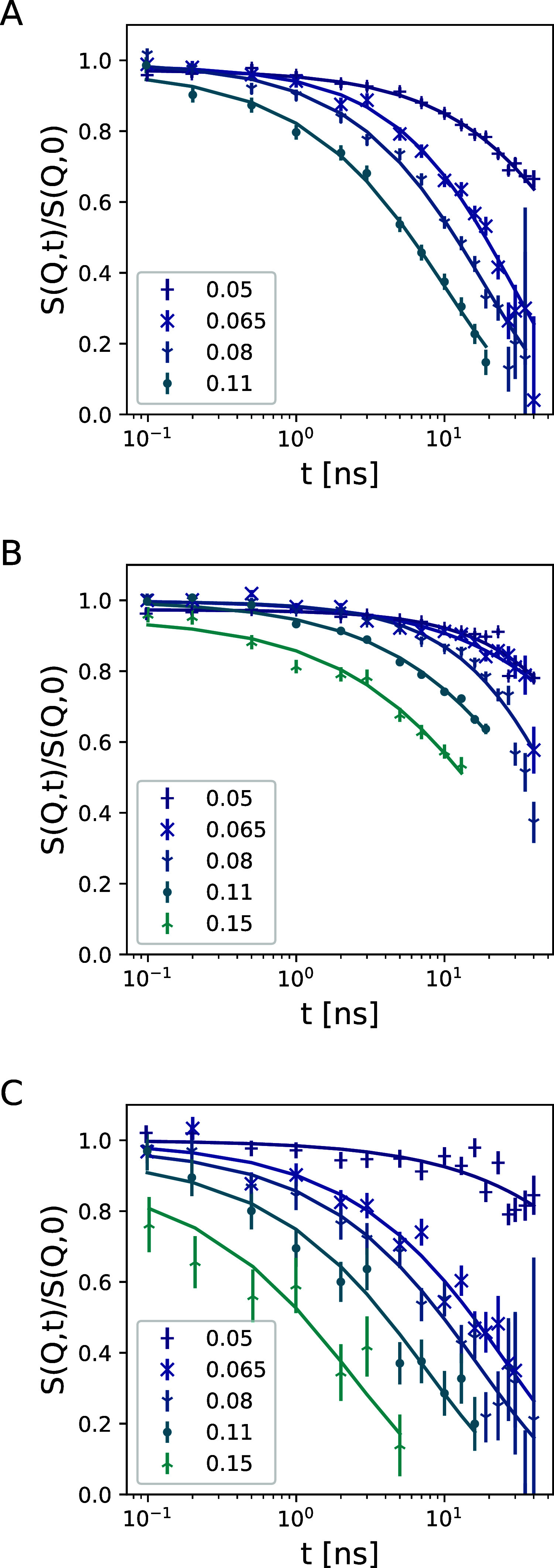
Intermediate scattering functions of (A) PEO homopolymers in solution, (B) core contrast, and (C) chain contrast. The *Q*-values correspond to the points in Figure 5. The solid lines display fits to a stretched exponential.

**Figure 5:**
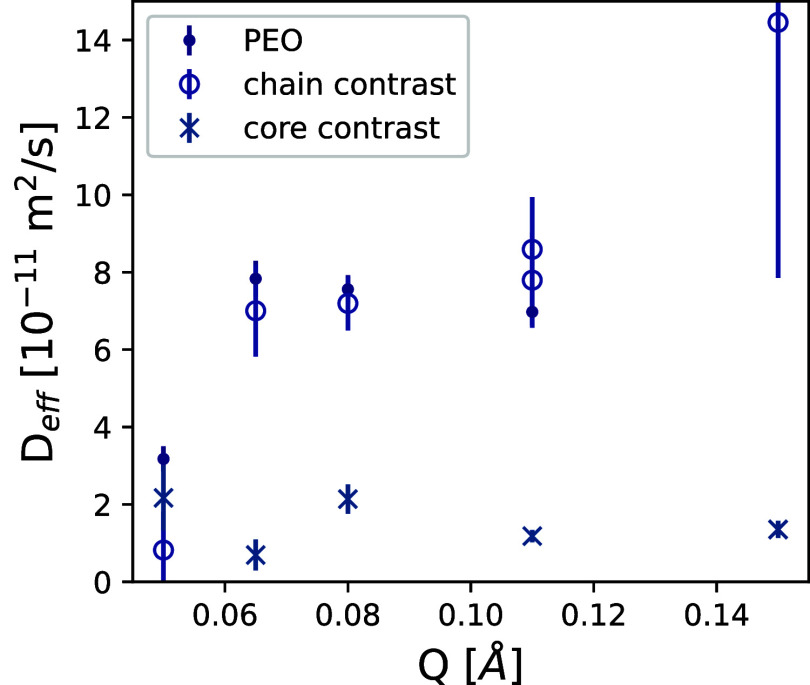
Effective diffusion constants *D*_eff_ for PEO homopolymer in solution, chain contrast, and core contrast. The dynamics of the PEO homopolymer and the chain contrast is very similar, indicating similar polymer-like mobility, with significantly slower dynamics obtained for the core contrast, where the connected C_16_ cores can only slowly diffuse as they are constrained by other cores and connecting chains.

The dynamics of the core is much slower, as is immediately visible in Figure 4B displayed in the results from the “core contrast”. It also shows a strong deviation from *Q* ^2^-dependence of the relaxation rate, indicating a very slow component outside the NSE Fourier time window. Nevertheless, a significant diffusion component can be observed.

The expected diffusion for a solvated chain (ref^36^ p. 130) is *D* = 0.196 *k*_B_ *T*/(*ηR*) = 7.4 × 10^–11^ m^2^/s for a chain with *R* = 10.8 × 10^–9^ m and a solvent viscosity η = 0.001 kg/(m s). This is in the right regime of the obtained effective diffusion constant. The slight increase in *Q* of the effective diffusion indicates Zimm dynamics with a *Q* ^3^-dependence instead of the *Q* ^2^-dependence, which already was anticipated from the curve shape of *S*(*Q*,*t*) with the stretching exponent β = 0.84.

For obtaining further insight, an analysis with the full Zimm model was applied. The fits to the full model are shown in the Supporting Information. The end-to-end distance *R*_ee_ = 10.8 nm was taken, and the viscosity was the only fitting parameter. The full Zimm model as described, e.g., in^40^ has been applied with a sum over all modes. The simultaneous fit of the full Zimm model for the PEO in solvent gives a good agreement for all available *Q*-values, with a viscosity of (1.8 ± 0.06) × 10^–3^ kg/(m s). The same fit procedure can also be applied to the gel sample in chain contrast. A value of 9.8 nm has been used, in agreement with the SANS data. The simultaneous fit again gives good agreement to the data from the chain contrast sample. The viscosity as the only fitting parameter determined in this way is (1.3 ± 0.07) × 10^–3^ kg/(m s). This is slightly lower than for the pure PEO homopolymer in solution and close to the viscosity of deuterated water, indicating that the chains behave as ideal polymers in the solvent obeying the Zimm model. The slightly higher viscosity observed in the case of PEO homopolymer might indicate that the pure polymer in the solution is not fully swollen and deviates from an ideal Gaussian chain, while the gel-like structure of the chain network expands better in solution and is less compact than the pure PEO due to the connectivity. The higher compactness of the PEO might then result in a slightly higher “apparent local viscosity”, as it has been observed also in other polymers in a polar solvent, like PNIPAM microgels in water.^41^ Interestingly, the rather ideal Zimm behavior of the chain network indicates that the network structure of this gel does not affect the Zimm modes of the polymer chains. It is still flexible enough that there is no mode restriction. In line with the SANS results, which do not suggest any stretching, the chain ends are statistically not “fixed”. Instead, they exhibit fluctuations continuously, exiting and reentering the micellar cores so that the connectivity does not significantly impact the short-time chain dynamics.

The core contrast samples exhibit a much slower relaxation, which is due to the slow, diffusive motion of the C_16_ micellar cores. The mobility of the cores is constrained by the surrounding cores and connecting chains, giving rise to a cage-like dynamics where the terminal relaxation is only at very long times, e.g., in rheological experiments. A distinction between this slow, (quasi)­elastic contribution and the cage diffusion is difficult to separate in a fit analysis in NSE. However, the dynamics is reflected in the strong *Q*-dependence of the dynamics that roughly follows the structure factor (“de Gennes narrowing”). This becomes obvious when plotting the relaxation time with a slightly increased *Q-*resolution (different detector binning in the NSE experiment) together with the SANS intensity where the peaks are associated with the structure factor (core contrast from Figure 3A). Instead of taking the full 2D detector with an Δ*Q* of ± 0.02 Å^–1^, single detector stripes with a width of Δ*Q* ≃ 0.01 Å^–1^ have been selected around the structure factor peak.

Figure 6 shows this comparison with the effective relaxation time and the total SANS intensity plotted as a function of *Q*.

**Figure 6:**
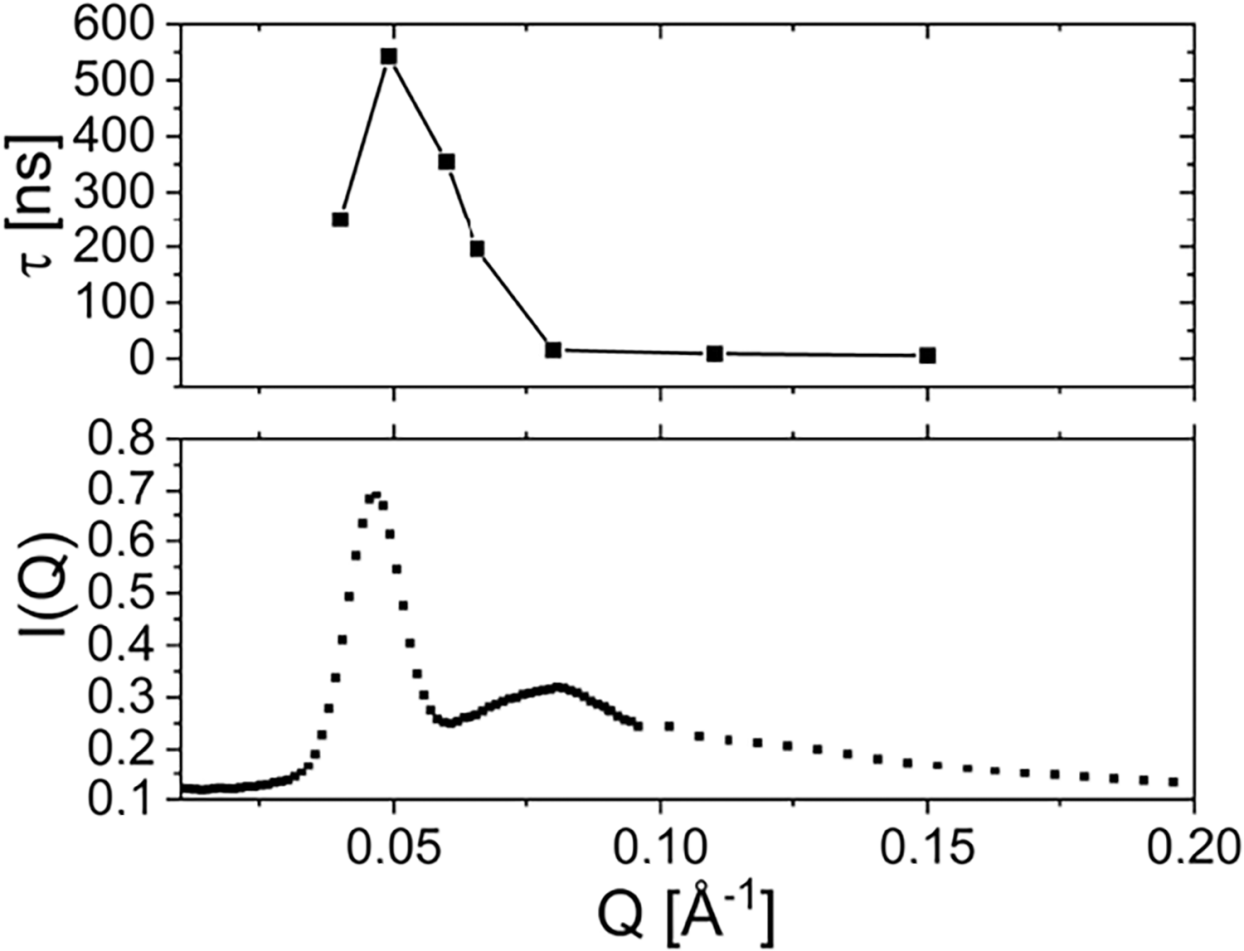
Structural slow down of the relaxation rate of the core. The relaxation time compared to the total scattering intensity from SANS. A slightly increased *Q*-resolution of the NSE data has been used.

## Conclusions

In this work, the aim was to investigate the dynamics of a self-healing polymer network in order. To this end, we have employed NSE, which is able to resolve the dynamics on the appropriate length scales of the polymers, revealing the internal chain dynamics and self-diffusion in a transient hydrogel networks spontaneously formed by telechelic polymers through self-assembly. The free PEO in solution shows Zimm dynamics with a slightly higher apparent viscosity, which might be attributed to a non-ideal Gaussian chain but rather a dense environment of the individual chain segments. Interestingly, we do not observe any change in the mode distribution or deviation due to constrained dynamics caused by transient cross-links. Under core contrast conditions, a slow relaxation can be observed, which can be attributed to the slow diffusive motion of the whole core, partly outside the NSE time window, and therefore appears as almost “elastic”. Possibly, the faster components observable at high *Q* are due to the disconnection and reconnection of core segments in the network and also from “mobile” segments at the surface of the cores. The single chain contrast of the gel exhibits Zimm dynamics, with the viscosity of the surrounding medium, i.e., the hydrodynamic interactions are very well captured in the dynamics. An influence of the confinement by the fixed ends at the cores is not visible, probably because the mobility of the cores themselves is high enough. The chain dynamics of the gel therefore seems to exhibit ideal Zimm dynamics. Deviations of the apparent viscosity, which have been observed in PNIPAAM microgels,^22^ for example, are not found here. The spanning of the polymers between the connecting core sites seems to prevent such a dense environment and possible interactions with neighboring chain segments, which might be the reason for the apparent higher viscosity visible in the PEO in solution. In conclusion, contrast variation highlighting selectively micellar cores and chain of the network provides very detailed insight into the decoupled dynamics, which is hard to separate using other methods. The study highlights the decoupling of the slow dynamics of the micellar cores, important for the rheological response, and the fast, relatively unconstrained chain dynamics, responsible for their “self-healing” properties. The results will be very useful for the fundamental understanding and applications of this type of self-healing hydrogels based on associative polymers.

## Supplementary Material



## Notes

Simultaneous Zimm fit in chain contrast with the solvent viscosity as the only fitting parameter; *Q*-values are (from top to bottom) 0.065, 0.08, 0.11, and 0.15 °A–1; size exclusion chromatograms of d-C16-h-PEO10-d-C16 with different contents of the parent diblock d-C16-h-PEO5, and form factor analysis of the free polymer chains and contrast matched chains belonging to the hydrogels (PDF)
