# Supplementary material for: Structure and Chain Dynamics of Self-Healing Telechelic Polymer Networks
Source: Macromolecules. 2025 Sep 2;58(18):9754–62. doi: 10.1021/acs.macromol.5c01216 (PMC12462249; doi:10.1021/acs.macromol.5c01216)
Supplement: Supplementary file 1 [file ma5c01216_si_001.pdf]

Supplementary Information: Structure and Chain Dynamics of Self-healing Telechelic Polymer Networks

Reidar Lund,[*,†] Lutz Willner,[¶] and Olaf Holderer[§]

†*Department of Chemistry, University of Oslo, Postboks 1033 Blindern, 0315 Oslo , Norway*

‡*Hylleraas Centre for Quantum Molecular Sciences, University of Oslo, Postboks 1033 Blindern, 0315 Oslo , Norway*

¶*Jülich Centre for Neutron Science (JCNS-1), Forschungszentrum Jülich GmbH, 52425 Jülich, Germany*

§*Jülich Centre for Neutron Science (JCNS) at Heinz Maier-Leibnitz Zentrum (MLZ), Forschungszentrum Jülich GmbH, 85747 Garching, Germany*

E-mail: reidar.lund@kjemi.uio.no

SANS analysis of chain conformation

To analyse the chain conformation in detail, the data were analysed using three different form factor; one for "ideal chains" following Gaussian statistics (Debye function), and two different describing chains with excluded volume interactions; Beaucage and Generalized Debye equations. All three models are described in the main document with the resulting fit parameters given in Table S1.

The fit analysis is given below in Figure S1:

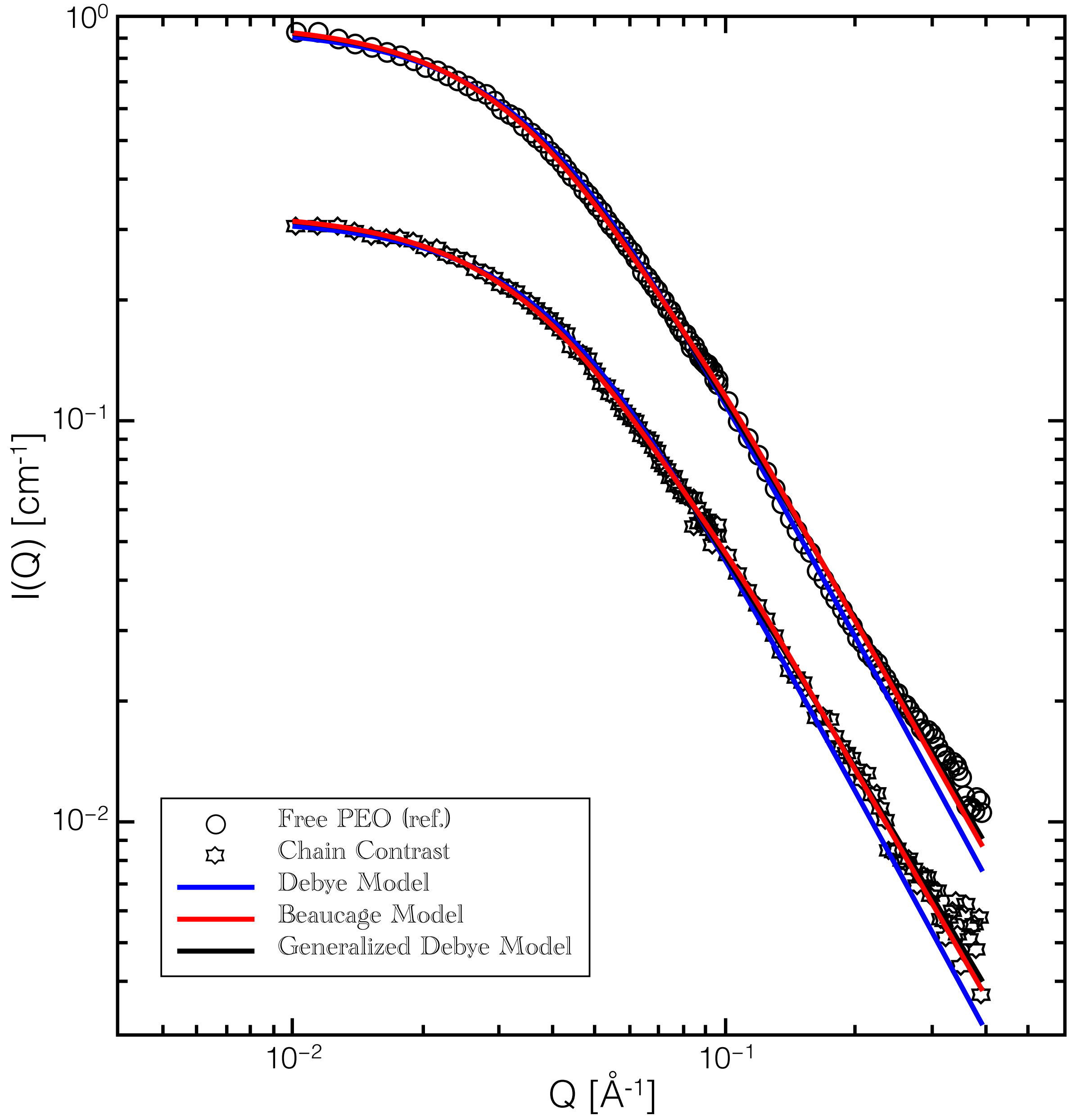

Figure 1: Form factor analysis of the free polymer chains and contrast matched chains belonging to the hydrogels

Table 1: Fit results SANS form factor analysis

Sample	R_g/Å	d_f	ν^*
Free Chain (Debye)	40 ± 3	2	0.5
Free Chain (Beaucage)	44 ± 3	1.9 ± 0.1	0.52
Free Chain (Gen. Debye)	44 ± 3	1.9 ± 0.1	0.53
Chain Contrast (Debye)	36 ± 2	2	0.5
Chain Contrast(Beaucage)	40 ± 2	1.9 ± 0.1	0.54
Chain Contrast(Gen. Debye)	39 ± 2	1.8 ± 0.1	0.55

Full Zimm Fits

The simplified fit with a stretched exponential function already revealed the characteristics of Zimm dynamics with the right stretching exponent and a Q^3-dependence of the relaxation rate (i.e. the diffusion has a linear dependence on Q). In the following, the simultaneous fit with the full Zimm model is shown, which also shows that this model describes well the data.

The NSE data of the PEO in solution and the single chain contrast have been fitted also with the full Zimm model (see e.g.)[1])

$$S(Q,t)/S(Q,0) = e^{-D_{CM}q^2t}/N \sum_{n,m}^{N} \exp\left(\frac{-q^2 B(m,n,t)}{6}\right) \tag{1}$$

with

$$B(m,n,t) = (n-m)^{2\nu} l^2 + \sum_{p=1}^{p_{max}} \frac{1}{p^{2\nu+1}} \cos\Big(\pi pn/N\Big) \cos\Big(\pi pm/N\Big) \left[1 - e^{-t/\tau_p}\right] \tag{2}$$

where the modes p with the Zimm time τ_p up to p_{max} of a polymer with N beads and segmental length $l = R_e/N^\nu$ are summed up. D_{CM} is the center-of-mass diffusion. From SANS, $R_g = 44$ and 39 Åwere obtained. The center-of-mass diffusion of a polymer chain is $D_{CM} = 0.196 k_B T/R_e/\eta$ with the viscosity η, the mode relaxation time $\tau_p = \eta R_e^3/(\sqrt{3\pi} k_B T) p^{-3\nu}$. Fitting has been done with $p_{max} = 20$, which is largely sufficient for capturing the main con-

tributing modes. The fitting parameter is then the viscosity, which indicates, if deviations from the solvent viscosity occur, if additional or stronger friction to the solvent is apparent.

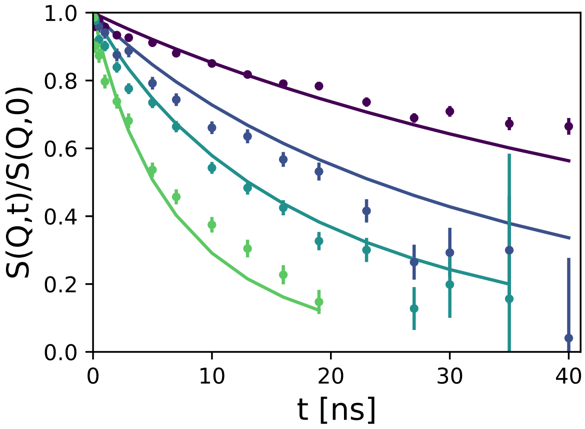

Figure 2: Simultaneous Zimm fit of PEO in solution with the solvent viscosity as only fitting parameter. Q-values are (from top to bottom) 0.065, 0.08, 0.11, 0.15 $Å^{-1}$

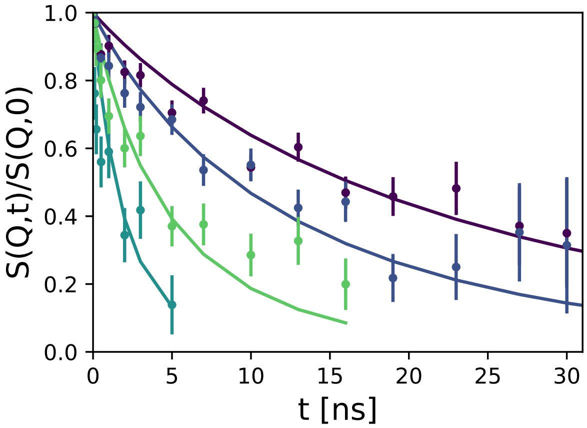

Figure 3: Simultaneous Zimm fit in chain contrast with the solvent viscosity as only fitting parameter. Q-values are (from top to bottom) 0.065, 0.08, 0.11, 0.15 $Å^{-1}$

Polymer characterisation

The polymers d-C_{16}-h-PEO10-d-C_{16} and h-C_{16}-d,h-PEO10-h-C_{16} were analyzed by size exclusion chromatography. Figure 4 shows the differential refractive index curves for the two polymers. The elution volumes are almost identical, indicating equal molecular volumes of the two polymers. In addition to the main peaks, a small shoulder appears at larger elution volumes arising from uncoupled diblock material which could not be entirely removed by fractionation.

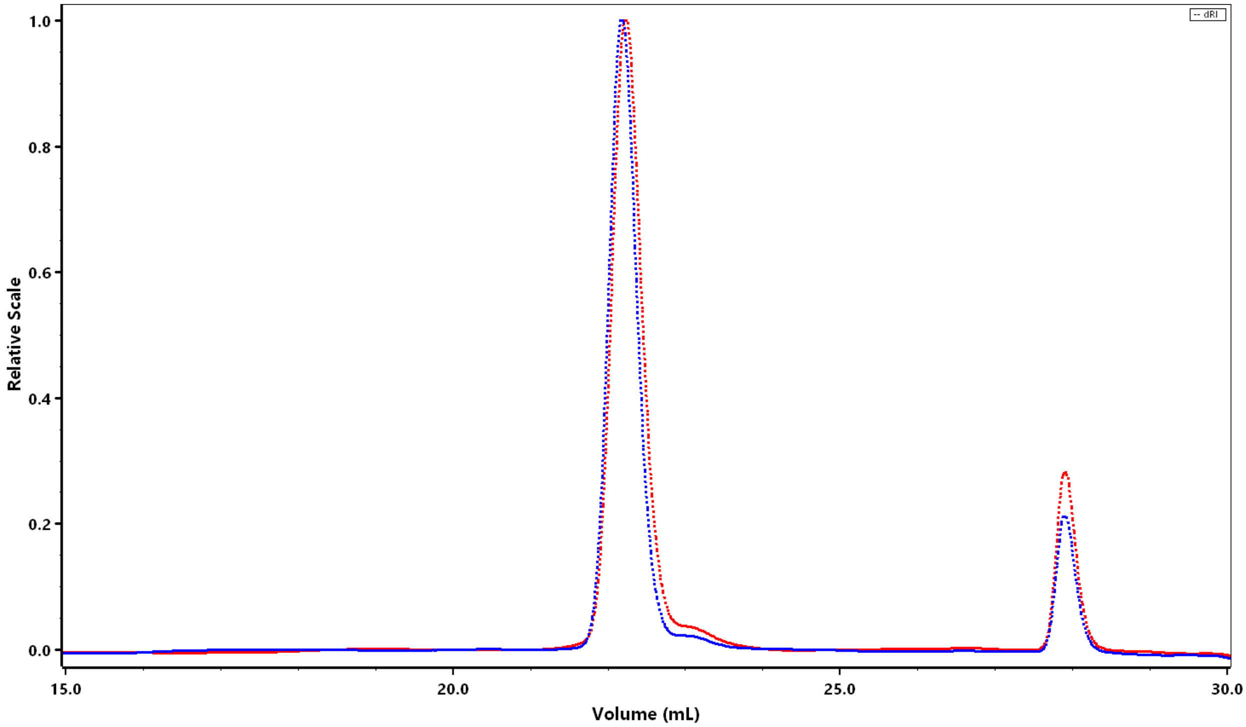

Figure 4: Size exclusion chromatograms of d-C_{16}-h-PEO10-d-C_{16} (red) and h-C_{16}-d,h-PEO10-h-C_{16} (blue). Signals at ≈ 28mL belong to octadecanol used as flow rate marker. The data were recorded with a chromatic set-up consisting of autosampler, isocratic pump (Agilent Technologies, Series 1260 infinity), column oven, refractive index detector, 18 angle light scattering detector (Wyatt Technologies, Optilab T-rex and Dawn Heleos-II), and three Agilent PlusPore GPC columns with a continuous pore size distribution. A mixture of tetrahydrofuran, N,N-dimethylacetamide , and acetic acid was taken as eluent. The measurements were conducted at 50°C at a flux rate of 1 mL min^{-1}. For a better visibility only the refractive index traces are shown in the figure.

The yield of fractionation of d-C_{16}-h-PEO10-d-C_{16} was too low to be able to perform neutron scattering experiments. Therefore, we have used combined fractions which still

contained 19% d-C_{16}-h-PEO5 diblock material. The corresponding SEC data are shown in Figure 5.

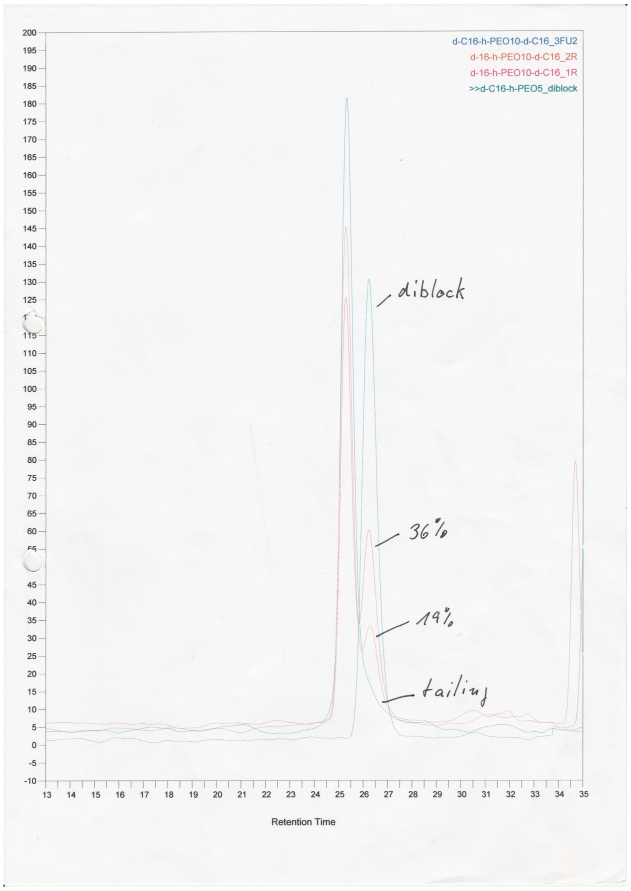

Figure 5: Size exclusion chromatograms of d-C_{16}-h-PEO10-d-C_{16} with different content of the parent diblock d-C_{16}-h-PEO5. The fraction with 19% residual diblock has been used for the experiments. The SEC data were collected with a Polymer Laboratories Agilent PL-GPC220 High Temperature System. Three Agilent PlusPore GPC columns with a continuous pore size distribution have been used and THF/DMA 84:16 v/v as solvent at a flux rate of 1 mL min^{-1}.

References

(1) Ameseder, F.; Radulescu, A.; Holderer, O.; Falus, P.; Richter, D.; Stadler, A. M. Relevance of internal friction and structural constraints for the dynamics of denatured bovine serum albumin. *The Journal of Physical Chemistry Letters* **2018**, *9*, 2469–2473.
